# Protein Delivery to Insect Epithelial Cells In Vivo: Potential Application to Functional Molecular Analysis of Proteins in Butterfly Wing Development

**DOI:** 10.3390/biotech12020028

**Published:** 2023-04-16

**Authors:** Yugo Nakazato, Joji M. Otaki

**Affiliations:** The BCPH Unit of Molecular Physiology, Department of Chemistry, Biology and Marine Science, Faculty of Science, University of the Ryukyus, Nishihara, Okinawa 903-0213, Japan

**Keywords:** endosome, green fluorescent protein, L17E, membrane-lytic peptide, pale grass blue butterfly, ProteoCarry, *Zizeeria maha*

## Abstract

Protein delivery to cells in vivo has great potential for the functional analysis of proteins in nonmodel organisms. In this study, using the butterfly wing system, we investigated a method of protein delivery to insect epithelial cells that allows for easy access, treatment, and observation in real time in vivo. Topical and systemic applications (called the sandwich and injection methods, respectively) were tested. In both methods, green/orange fluorescent proteins (GFP/OFP) were naturally incorporated into intracellular vesicles and occasionally into the cytosol from the apical surface without any delivery reagent. However, the antibodies were not delivered by the sandwich method at all, and were delivered only into vesicles by the injection method. A membrane-lytic peptide, L17E, appeared to slightly improve the delivery of GFP/OFP and antibodies. A novel peptide reagent, ProteoCarry, successfully promoted the delivery of both GFP/OFP and antibodies into the cytosol via both the sandwich and injection methods. These protein delivery results will provide opportunities for the functional molecular analysis of proteins in butterfly wing development, and may offer a new way to deliver proteins into target cells in vivo in nonmodel organisms.

## 1. Introduction

Genetic engineering is a major force in biotechnology with its wide applications to basic biology, medicine, and agriculture. Recent advancements in CRISPR/Cas9 genome editing ushered in a new era in biotechnology because this technology is applicable to a wide variety of organisms other than typical model organisms, such as the house mouse and the fruit fly [1,2]. However, no single method is perfect for diverse biological systems and applications. For example, genome editing often affects cellular differentiation before reaching the stage of interest and is often technically demanding in many nonmodel organisms. In the case of insects, gene transfer with baculoviral vectors can be an alternative approach [3,4,5,6,7], but viral toxicity and the period of time required for gene expression from the viral vectors may limit its use and interpretations.

Additionally, there has been a demand for the direct delivery of proteins or drugs to cells of interest. In the late twentieth century, liposome-based drug delivery systems have been tested [8]. Delivery liposomes are usually equipped with antibodies on their surface for cell targeting [8]. Furthermore, such liposomes are often combined with fusiogenic viral envelope proteins or their related synthetic proteins [8,9]. Direct protein delivery without liposomes was made possible by the introduction of protein transduction domains (PTDs) [10,11]. PTDs are also called cell-penetrating/permeable peptides (CPPs), which are derived from several membrane-crossing proteins [10,11,12]. In early protein transduction technology, proteins of interest were covalently linked with one of the PTDs [10,11,12]. Since delivered proteins are often confined within endosomes, protein release from endosomes to the cytosolic side has been an important technological objective [13,14]. In 2014, dfTAT, one of the PTDs, was shown to deliver proteins into the cytosol without covalent linkage to proteins [15].

However, the obstacle that proteins are often incorporated into a cell by endocytosis and are confined within endosomes has not been completely overcome. Several different peptides and their modified versions have been tested for more efficient endosomal escape [16,17,18,19,20,21,22,23,24,25,26,27,28,29,30,31,32,33,34]. Due to the technological demands for quick protein delivery to cells with fewer technical requirements, membrane-lytic peptides are receiving more attention from researchers in the twenty-first century. Protein delivery is often used for cultured cell lines in vitro, and in vivo protein delivery is still not very popular, although such studies already exist [17,20,25,28]. This may be partly due to physical barriers of the extracellular matrix, and accordingly, in vivo delivery systems often require specific considerations that are inherent to that biological system. Indeed, to our knowledge, studies on in vivo protein delivery to insect cells are scarce. However, it is worth noting that a direct injection of Cas9 ribonucleoproteins into the adult abdomen of cockroaches successfully delivered them into oocytes to obtain genome-edited progeny [35]. Thus, this genome editing study suggests that developing insect cells may accept foreign proteins relatively easily.

In the field of developmental biology of butterfly wings, genome-edited butterflies revealed functions of genes such as *Distal-less* and *Wnt* genes in color pattern determination [36,37,38,39]. However, genome editing is not the perfect solution to understanding molecular functions because the critical period of color pattern determination is just several hours after pupation [40,41]. To obtain conclusive results, researchers need to change molecular expression only during this time window. In reality, molecular expression may be changed throughout the developmental period in genome-edited butterflies, which may result in a possible misinterpretation of molecular functions. As a result of this limited window for molecular interference, baculovirus vectors were used previously, but this method has the problem of cellular toxicity. There is a reported case of a successful RNAi method for color pattern modifications in the swallowtail butterfly [42]. However, this study used electroporation [43], together with the forewing-lift method [44,45], which necessitates the use of a device specially built for this purpose. Establishing genetic lines with drug-inducible genes in the genome may be a solution, but these complex genetic manipulations and line maintenance are not practical for researchers using nonmodel organisms.

In this study, we investigated the possibility of in vivo protein delivery to insect epithelial cells in pupal butterfly wings. The butterfly wing system is an ideal system to evaluate the efficacy of in vivo protein delivery because epithelial cells can be easily accessed, treated, and observed in real time in vivo during development. The pupal wing epithelial tissues are accessible from outside after the forewing-lift operation, which was first invented in 2009 [44] and can be used for real-time imaging [45]. The epithelium consists of a single layer of immature scale and socket cells [46]. Previously, sodium tungstate was applied by this method [47]. Fluorescent dyes can be applied to stain organelles of epithelial cells in vivo [46,48,49]. In addition to this topical application, abdominal injection may be able to deliver molecules into epithelial cells. Methodologically, it was not until 1998 that the abdominal injection of chemicals into butterfly pupae was performed [50]. This simple injection into pupae has been used to study color pattern modifications since then. In a previous study, injected FB28, a fluorescent dye, was demonstrated to reach the apical extracellular space just above the epithelium [51].

In the present study, we used both topical and systemic methods for protein delivery: the sandwich method, in which liquid samples were sandwiched between the forewing and the hindwing immediately after pupation, and the injection method, in which liquid samples were injected into the abdomen. Using these two application methods, we examined whether green fluorescent protein (GFP) and orange fluorescent protein (OFP) are delivered into cells using fluorescence confocal microscopy. Additionally, we used antibodies conjugated with fluorescent dyes. Antibodies can potentially be used to inhibit protein functions inside cells. These proteins were applied without any protein delivery reagent, with L17E [18,22,23,30,32] or with ProteoCarry [52]. L17E and ProteoCarry are membrane-lytic peptides used for protein delivery. We chose L17E and ProteoCarry because they are now commercially available and easy to use for potential users outside the protein delivery field.

## 2. Materials and Methods

### 2.1. Butterfly Rearing

The present study used the pale grass blue butterfly *Zizeeria maha* to test protein delivery. The butterflies were reared according to methods of previous studies [53,54]. The adult females of this butterfly were caught in the Nishihara Campus of the University of the Ryukyus. The eggs were collected on its host plant, the creeping wood sorrel *Oxalis corniculata*, under laboratory conditions. Larvae hatched from these eggs were reared with the host plant leaves in plastic containers until pupation at ambient temperatures (approximately 26 °C). Occasionally, we also used adult females from these pupae to obtain eggs in the next generation. To avoid sibling crosses, adult males were caught in the field and crossed with virgin females reared in the laboratory.

### 2.2. Experimental Operations

We used the sandwich method and the injection method for protein delivery in this study. The sandwich method was an application of the forewing-lift method [44,45], and was first performed in the pale grass blue butterfly in a previous imaging study [49]. Immediately after pupation, the left forewing was lifted under a stereomicroscope using forceps. A 4-microliter droplet (containing a delivery reagent and a protein of interest such as GFP/OFP or an antibody) was first placed on the surface of the dorsal hindwing and was sandwiched between the ventral forewing and the dorsal hindwing. After incubation for one or two hours, the surface of the wing tissue was washed with phosphate buffered saline (PBS), and the fluorescent dyes were sandwiched similarly for one hour. Then, the wing tissue was washed with PBS. The surface of the ventral forewing and the dorsal hindwing were placed on a thin glass plate and covered with a piece of plastic wrap to prevent water evaporation. The ventral forewing was subjected to fluorescence laser-scanning confocal microscopy. These operations were started within 20 min post-pupation. The pupation timing was occasionally adjusted by placing prepupae in an incubator set at 15 °C.

For the injection method, injection of a protein of interest and a delivery reagent was performed into the abdomen more than two hours post-pupation using an Ito microsyringe (Fuji, Shizuoka, Japan). This injection process was executed after the dye loading process through the sandwich method for one hour, and the wing tissue was placed on a glass plate as described above. The injection volume was 0.5 μL per individual.

### 2.3. Fluorescent Proteins, Antibodies, and Delivery Reagents

Recombinant enhanced GFP (eGFP) expressed and purified from *Escherichia coli* (product code: NBP2-34923) was purchased from Novus Biologicals (Centennial, CO, USA). This is a single nonglycosylated polypeptide chain of 26.9 kDa with 239 amino acid residues that was first identified and purified from the jellyfish *Aequorea victoria*. The eGFP contains S65T and F64L mutations. In the present study, the eGFP used here was called “GFP” for simplicity. On the other hand, OFP is an orange/red fluorescent protein sold as OFPSpark (product code: 69002-S08E) from Sino Biological (Beijing, China). It is a 26.4-kDa protein derived from DsRed from *Discosoma* sp. containing 231 amino acid residues. In the present study, the OFPSpark used here was called “OFP” for simplicity. Since OFP has relatively high pH stability, it may be able to fluoresce within a lysosome. The final concentration of GFP/OFP was 0.41 nM (at the 8:1 volumetric ratio for ProteoCarry; see below) or 1.85 nM (at the 1:1 volumetric ratio; see below).

Anti-HSP (heat shock protein) antibody labeled with FITC (fluorescein isothiocyanate) (mouse monoclonal) against human HSP70 expressed in *E. coli* (product code: SMC-162D-FITC) was purchased from StressMarq Biosciences (Cadboro Bay Village, BC, Canada). The final concentration of this antibody used in the present study was 25 μg/mL (1:1), 111 μg/mL (8:1), or 250 μg/mL (1:1). Anti-tubulin antibody (clone YL1/2) labeled with FITC (rat monoclonal) against α-tubulin purified from *Saccharomyces cerevisiae* (product code: NB600-506F) was purchased from Novus Biologicals. The same antibody labeled with DyLight 550 (product code: NB600-506R) was also purchased from the same manufacturer. The final concentration of this antibody used in the present study was 25 μg/mL (1:1), 66.7 μg/mL (8:1), 250 μg/mL (1:1), or 300 μg/mL (1:1). Anti-*Drosophila* axons antibody labeled with Alexa Fluor 488 (product code: sc-53018 AF488) was purchased from Santa Cruz Biotechnology (Dallas, TX, USA). This is a mouse monoclonal antibody raised against axons of the central nervous system in *Drosophila*. The final concentration of this antibody used in the present study was 22.2 μg/mL (8:1) or 100 μg/mL.(1:1). For a blocking procedure, the epithelial tissue was first incubated with normal mouse IgG (product code: sc-2025) (Santa Cruz Biotechnology) at a final concentration of 25 μg/mL in PBS for 30 min before the protein delivery procedures.

L17E (IWLTALKFLGKHAAKHEAKQQLSKL-amide) [18] was chemically synthesized using the Fmoc method (GenScript Japan, Tokyo, Japan) with a purity of 99.6%. The product was confirmed by LC-MS spectrograms. L17E was originally a spider toxin (M-lycotoxin) that was improved for endosomolytic activity [18]. L17E Cytosolic Delivery Peptide is now manufactured by Peptide Institute (product code: 3409-v), Osaka, Japan and sold from FUJIFILM Wako Chemicals, Osaka, Japan (product code: 335-34091). Regarding the concentration of L17E, we primarily used 40 μM and 40 mM (before mixing with other solutions) for the sandwich and injection methods, respectively, based on a previous study [18]. To make the final concentration, we used a 1:1 volumetric ratio of L17E to a protein of interest. We tried up to 10,000 times L17E, but we observed no clear improvement.

Another endosomolytic peptide regent, ProteoCarry (product code: FDV-0015) [52], was purchased from Funakoshi (Tokyo, Japan). ProteoCarry is a proprietary reagent with little publicly available information on its structure. Functional information from the manufacturer is available at https://www.funakoshi.co.jp/exports_contents/80968 [52] (accessed on 29 November 2022). The ratio of ProteoCarry to a protein of interest for the sandwich method was 1:1 or 8:1 in volumetric ratio. The ratio of ProteoCarry to a protein of interest for the injection method was 8:1.

### 2.4. Fluorescent Dyes and Confocal Imaging

For confocal imaging of the epithelial cells, we used Hoechst 33342 (product code: 346-07951) (Dojindo Molecular Technologies) for nuclear staining, MitoRed (product code: R237) (Dojindo Molecular Technologies) for mitochondrial staining, LysoTracker Red DND-99 (product code: L7528) (Thermo Fisher Scientific, Tokyo, Japan) for lysosomal staining, and BODIPY FL C_5_-ceramide complexed to BSA (product code: B22650) (Thermo Fisher Scientific) for staining membranous structures such as the plasma membrane, endoplasmic reticulum, Golgi apparatus, and vesicles. The fluorescent dyes were diluted with dimethyl sulfoxide (DMSO). The final concentrations used in the present study were as follows: Hoechst 33342 (89.0–236.0 μM), MitoRed (10.7 μM), LysoTracker Red (5.5 μM), and BODIPY FL C_5_-ceramide (38.0–50.0 μM).

For confocal microscopy of the wing epithelium of the pale grass blue butterfly, we followed a previous study [49]. Briefly, we employed a Nikon A1^+^ ECLIPSE Ti confocal microscope system (Tokyo, Japan), as described in previous research. Confocal images were acquired and processed for cross-sectional and three-dimensional reconstructions using NIS-Elements AR 4.20.00 64-bit (Nikon). The excitation wavelengths by solid lasers were 405 nm, 488 nm, and 561 nm, for which the filtered emission wavelengths were 425–475 nm, 500–550 nm, and 570–620 nm, respectively. The software’s zoom functions (20 × 4, 20 × 5, and 100 × 2) were often employed. From the surface of the wing to deeper levels, many optical sections were obtained with 0.4–0.5-μm steps, which were used for three-dimensional reconstruction of the epithelial sheet. The optical sections were obtained from the surface (set at 0.00 μm) to a depth of 18.10 μm to 68.00 μm.

### 2.5. Endosome versus Cytosol Estimates

We qualitatively estimated whether a protein of interest was delivered only to cellular vesicles (i.e., endosomes and lysosomes) or to the cytosol, based on confocal images of fluorescent proteins and dyes. We assumed that endosomes were readily fused with lysosomes, which could be detected by LysoTracker Red. Thus, LysoTracker-positive signals were considered noncytosolic. For LysoTracker-negative signals, diffuse staining of cells was considered cytosolic. We assumed that in the most extensive cases, the cytosol would be entirely positive for a protein of interest, delineating the cellular shape. Cellular shapes were confirmed with BODIPY staining. When LysoTracker Red was not used, we estimated cytosolic staining based on the BODIPY staining pattern.

We did not perform quantitative analyses on the confocal images for signal colocalization for the following reasons to avoid misleading implications. First, due to an in vivo system, delivery efficiency varied from experiment to experiment, from individual to individual, and from area to area, even in a single individual. To circumvent this problem, we showed both the number of positive individuals (*n_p_*) and the total number of tested individuals (*n_t_*) in each experiment. Second, colocalization in this study mostly indicates a failure of cytosolic delivery. Rather, existence (or nonexistence) of diffuse cytosolic signals (not dotted signals) was the most important point of this study. Third, we used not only LysoTracker Red but also BODIPY to estimate cytosolic staining. Since BODIPY stains various membranous structures, colocalization analysis was not adequate for such experiments.

## 3. Results

### 3.1. Protein Delivery without Delivery Reagent

#### 3.1.1. Sandwich Method

**Figure 1:**
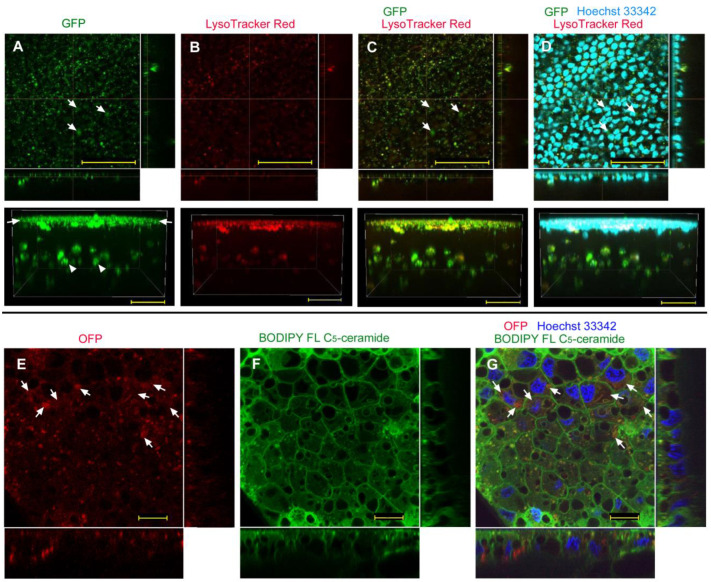
GFP/OFP application using the sandwich method without delivery reagent. Optical cross sections are shown at the right and bottom of the major plane panel. (**A**–**D**) A treated individual. The four panels (**A**–**D**) are images of the same visual field. In addition to the optical cross sections, a side view of a three-dimensional reconstruction image (apical side up) is shown at the bottom. Scale bars: 50 μm. (**A**) GFP. Arrows indicate apical GFP signals (also indicated in (**C**,**D**)). Arrowheads indicate basal GFP signals. (**B**) LysoTracker Red. (**C**) Merge of GFP and LysoTracker Red. (**D**) Merged GFP, LysoTracker Red, and Hoechst 33342 images. (**E**–**G**) Another treated individual. The three panels (**E**–**G**) are images of the same visual field. Scale bars: 10 μm. (**E**) OFP. Arrows indicate diffuse OFP signals (also indicated in (**G**)). (**F**) BODIPY FL C_5_-ceramide. (**G**) Merge of OFP, BODIPY FL C_5_-ceramide, and Hoechst 33342.

We first examined the possibility of GFP delivery using the sandwich method without any delivery reagent (*n_p_* = 2, *n_t_* = 4; the number *n_p_* hereafter indicates the number of individuals that successfully showed fluorescence-positive images, as shown in the figures; the number *n_t_* hereafter indicates the total number of individuals treated). GFP signals were observed as numerous dots, which were likely vesicles (i.e., endosomes and lysosomes) (Figure 1A, top). A three-dimensional image suggested that the major GFP signals were located at the apical portion of the epithelium (Figure 1A, bottom). Large GFP signal clusters at the basal portion may be macrophage-like hemocytes associated with the epithelium (Figure 1A, bottom). When the epithelium was stained simultaneously with LysoTracker Red (Figure 1B), we observed that most GFP-positive puncta colocalized with the red signals from lysosomes (Figure 1C), suggesting that most GFP molecules were incorporated into endosomes via endocytosis, and then into lysosomes. However, there seemed to be a small number of GFP signals that did not colocalize with the LysoTracker Red signals. Nuclear staining with Hoechst 33342 confirmed the presence of the nuclear layer of the epithelium, which was basically located at a similar depth to the GFP-positive layer within the epithelium (Figure 1D).

To clarify cellular outlines, we next employed OFP (Figure 1E) with BODIPY FL C_5_-ceramide for membranous structures (Figure 1F) and with Hoechst 33342 for nuclei (Figure 1G) and observed their fluorescent signals in higher magnification images (*n_p_* = 8; *n_t_* = 11). As in the case of GFP, OFP signals were dotted, possibly in endosomes and lysosomes (Figure 1E). However, diffuse OFP signals were also observed in many cells, which could be cytosolic signals (Figure 1E). Plasma membranes were well defined, clarifying cellular outlines (Figure 1F). A merged image of OFP and BODIPY indicated that some cells were stained red in their cytosol (Figure 1G), suggesting that epithelial cells may incorporate foreign proteins without any delivery reagent at least to some extent.

**Figure 2:**
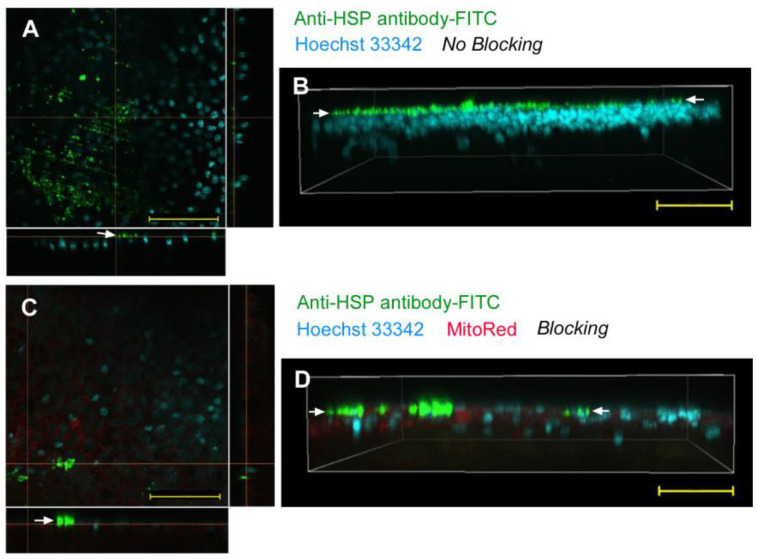
Application of anti-HSP antibody (conjugated with FITC) with the sandwich method without delivery reagent. Arrows indicate a layer of FITC signals on top of the nuclear and mitochondrial signals. Optical cross sections are shown at the right and bottom of (**A**,**C**). Scale bars: 50 μm. (**A**) Merge of FITC and Hoechst 33342 without blocking. (**B**) Three-dimensional reconstruction of optical sections (apical side up) showing a merge of FITC and Hoechst 33342 without blocking. (**C**) Merge of FITC, MitoRed, and Hoechst 33342 with blocking. (**D**) Three-dimensional reconstruction of optical sections (apical side up) showing a merge of FITC, MitoRed, and Hoechst 33342 with blocking.

Using the sandwich method, we applied an anti-HSP antibody (labeled with FITC) to the epithelial tissue without any delivery reagent. FITC signals were located extracellularly above the nuclear layer of cells in the epithelium, suggesting that the antibody could not be delivered into cells (*n_p_* = 0; *n_t_* = 2) (Figure 2A,B). To reduce nonspecific binding of antibodies to cuticle or extracellular matrix molecules, we introduced a blocking procedure; the wing tissue was first incubated with normal IgG. This blocking procedure did not improve the delivery efficiency at all; FITC signals were located above the epithelium (*n_p_* = 0; *n_t_* = 3) (Figure 2C,D). Another antibody, anti-tubulin antibody (labeled with FITC), did not change the results (*n_p_* = 0; *n_t_* = 2). These results showed that antibodies were not delivered into cells without delivery reagent in the sandwich method.

#### 3.1.2. Injection Method

**Figure 3:**
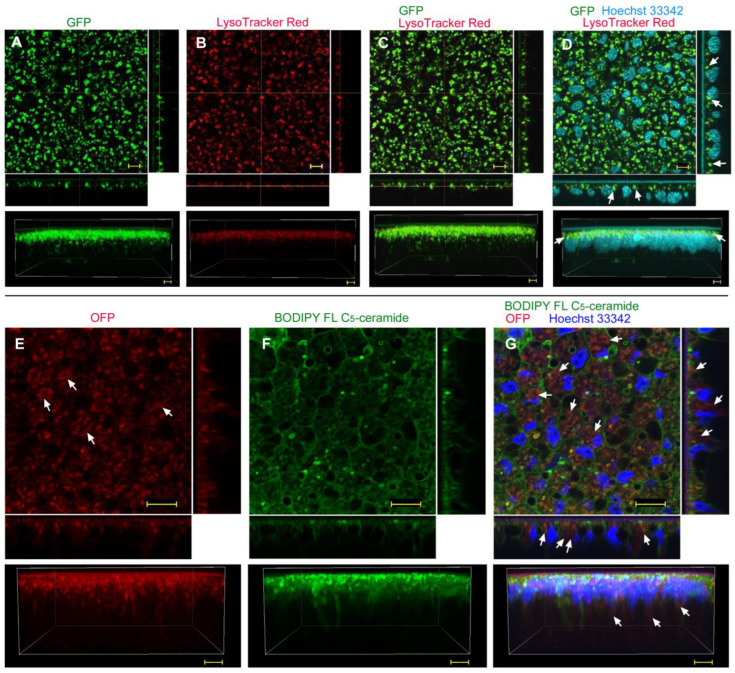
GFP/OFP application using the injection method without delivery reagent. Optical cross sections are shown at the right and bottom of the major plane panel. In addition, a side view of a three-dimensional reconstruction image (apical side up) is shown at the bottom. (**A**–**D**) A treated individual. Four panels (**A**–**D**) are images of the same visual field. Scale bars: 5 μm. (**A**) GFP. (**B**) LysoTracker Red. (**C**) Merge of GFP and LysoTracker Red. (**D**) Merged GFP, LysoTracker Red, and Hoechst 33342 images. Arrows indicate some GFP-positive and LysoTracker-positive endosomes located at the apical portion of nuclei. (**E**–**G**) Another treated individual. Three panels (**E**–**G**) are images of the same visual field. Scale bars: 10 μm. (**E**) OFP. Some diffuse OFP signals are indicated by arrows. (**F**) BODIPY FL C_5_-ceramide. (**G**) Merge of OFP, BODIPY FL C_5_-ceramide, and Hoechst 33342. Arrows indicate some diffuse OFP signals.

Abdominal injection of GFP without any delivery reagent resulted in similar results to the sandwich method; numerous GFP-positive dots were observed (*n_p_* = 4; *n_t_* = 4) (Figure 3A). Most GFP signals overlapped with the LysoTracker Red signals (Figure 3B,C), suggesting that GFP was taken up via endocytosis and then transferred to lysosomes. However, a three-dimensional image showed extensive GFP signals (Figure 3A, bottom); there was a possibility of cytosolic delivery in some cells. Nuclear staining with Hoechst 33342 clearly showed that GFP signals were observed in the apical portion of the epithelium (Figure 3D), suggesting that GFP was first transferred from the injection site to the apical extracellular side, and was incorporated into cells by endocytosis.

We then employed OFP (Figure 3E) and BODIPY FL C_5_-ceramide for membranous structures to visualize the plasma membrane and individual cells (Figure 3F), together with Hoechst 33342 for nuclear staining (*n_p_* = 4; *n_t_* = 4) (Figure 3G). As in the case of GFP, there were numerous OFP-positive dots within cellular compartments delineated by BODIPY. It is likely that these OFP molecules were in lysosomes. However, the epithelial cells were diffusely stained with OFP (Figure 3E), and some OFP signals were observed in the deeper portion of the epithelial cells (Figure 3E,G). Thus, some OFP molecules appeared to be delivered into the cytosol. We also confirmed that OFP was likely taken up by cells from the apical side, although OFP was injected into the abdomen.

**Figure 4:**
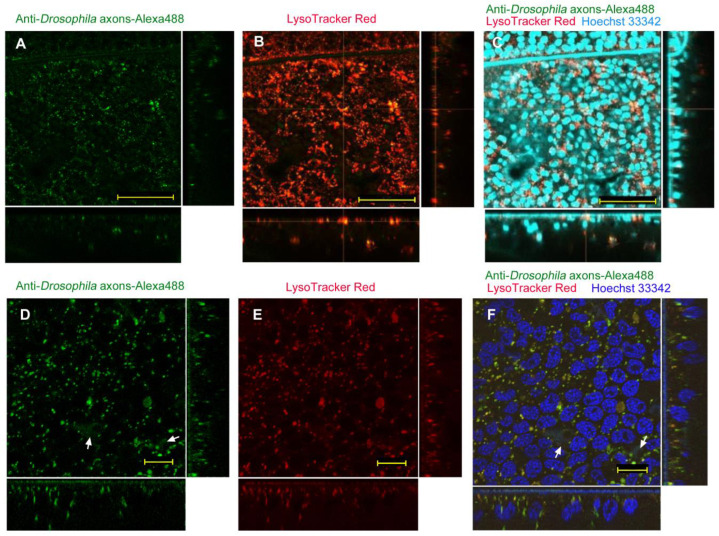
Application of anti-*Drosophila* axons antibody (conjugated with Alexa 488) using the injection method without delivery reagent. Optical cross sections are shown at the right and bottom of the major plane panel. Scale bars: 50 μm. (**A**–**C**) A treated individual. Three panels (**A**–**C**) are images of the same visual field. (**A**) Alexa 488. (**B**) LysoTracker Red. (**C**) Merge of Alexa 488, LysoTracker Red, and Hoechst 33342. (**D**–**F**) Another treated individual. Three panels (**D**–**F**) are images of the same visual field. Arrows indicate a few diffuse Alexa 488 signals. Scale bars: 10 μm. (**D**) Alexa 488. (**E**) LysoTracker Red. (**F**) Merge of Alexa 488, LysoTracker Red, and Hoechst 33342.

Abdominal injection of anti-*Drosophila* axons antibody (labeled with Alexa 488) without any delivery reagent showed dotted Alexa 488 signals in epithelial cells (*n_p_* = 6; *n_t_* = 6) (Figure 4A). The Alexa-positive layer appeared to overlap with LysoTracker Red signals entirely (Figure 4B,C). It appeared that protein injections generally activated high levels of lysosomal development, judging from the extensive LysoTracker Red signals (Figure 4B). Overlapping of the Alexa and LysoTracker signals was confirmed using higher magnification images in different sets of individuals (*n_p_* = 2; *n_t_* = 4) (Figure 4D–F). Under higher magnification, it seemed that almost no cytosolic delivery of anti-*Drosophila* axons antibody was achieved without delivery reagent, although rare, weak, and diffuse Alexa 488 signals were observed (Figure 4D,F). We also employed a different antibody, anti-HSP antibody (labeled with FITC), and obtained similar results with numerous dots overlapping with LysoTracker Red (*n_p_* = 3; *n_t_* = 8), confirming the negative results of the anti-*Drosophila* axons antibody.

### 3.2. Protein Delivery with L17E Delivery Reagent

#### 3.2.1. Sandwich Method

**Figure 5:**
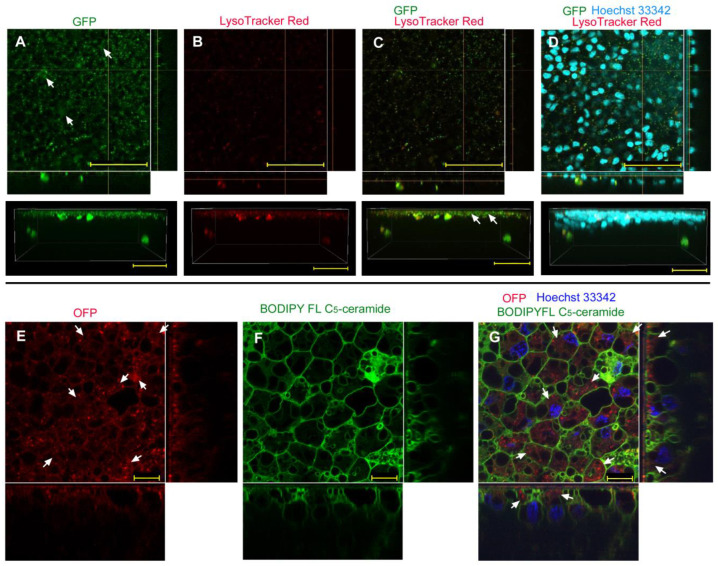
GFP/OFP application using the sandwich method with L17E. Optical cross sections are shown at the right and bottom of the major plane panel. In addition, a side view of a three-dimensional reconstruction image (apical side up) is shown at the bottom of (**A**–**D**). (**A**–**D**) A treated individual. Four panels (**A**–**D**) are images of the same visual field. Scale bars: 50 μm. (**A**) GFP. Arrows indicate a few diffuse GFP signals. (**B**) LysoTracker Red. (**C**) Merge of GFP and LysoTracker Red. Arrows indicate GFP-positive signals without overlap with LysoTracker Red. (**D**) Merged GFP, LysoTracker Red, and Hoechst 33342 images. (**E**–**G**) Another treated individual. Three panels (**E**–**G**) are images of the same visual field. Arrows indicate diffuse OFP signals. Scale bars: 10 μm. (**E**) OFP. (**F**) BODIPY FL C_5_-ceramide. (**G**) Merge of OFP, BODIPY FL C_5_-ceramide, and Hoechst 33342.

Using the sandwich method, GFP was incorporated into cells with L17E (*n_p_* = 4; *n_t_* = 4) (Figure 5A). Most GFP signals appeared to overlap with LysoTracker Red signals (Figure 5B,C), but some GFP signals, although weak, appeared to be diffuse without overlap, suggesting that some GFP molecules may have been in the cytosol (Figure 5C). Nuclear staining with Hoechst 33342 indicated that GFP signals were present in the same layer as nuclei (Figure 5D).

We also performed OFP delivery (Figure 5E) and BODIPY FL C_5_-ceramide staining (Figure 5F) at higher magnification (*n_p_* = 4; *n_t_* = 5). The OFP results with BODIPY were similar to the previous GFP results, showing dotted OFP signals inside cells, but importantly, many cells appeared to have cytosolic OFP signals (Figure 5E,G). Compared to the results without any delivery reagent, the L17E results may be as efficient or slightly more efficient in terms of cytosolic delivery of GFP/OFP.

**Figure 6:**
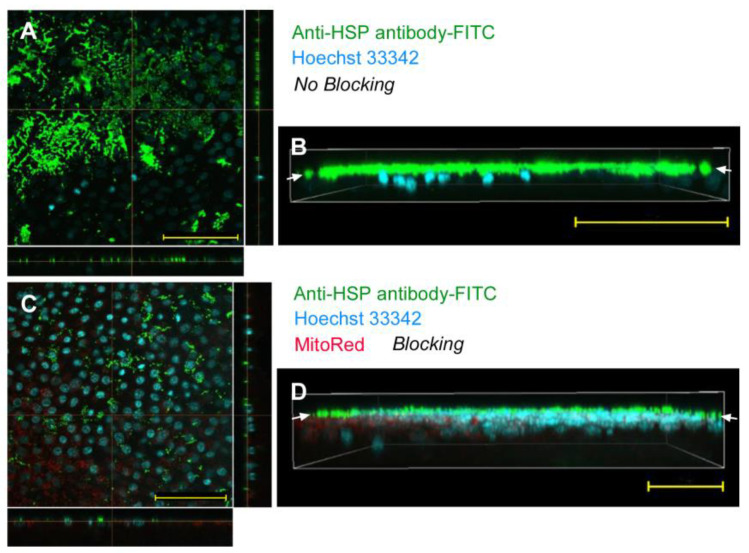
Application of anti-HSP antibody (conjugated with FITC) using the sandwich method with L17E. Optical cross sections are shown at the right and bottom of (**A**,**C**). Scale bars: 50 μm. (**A**) Merge of FITC and Hoechst 33342 without blocking. (**B**) Three-dimensional reconstruction of optical sections (apical side up) showing a merge of FITC and Hoechst 33342 without blocking. The FITC-positive layer is indicated by arrows. (**C**) Merge of FITC, MitoRed, and Hoechst 33342 with blocking. (**D**) Three-dimensional reconstruction of optical sections (apical side up) showing a merge of FITC, MitoRed, and Hoechst 33342 with blocking. The FITC-positive layer is indicated by arrows.

When anti-HSP antibody (labeled with FITC) was employed with L17E in the sandwich method, we were unable to detect any FITC signals from cells; the FITC signals were located just in the apical extracellular side above the Hoechst 33342 nuclear staining in the epithelium (*n_p_* = 0; *n_t_* = 6) (Figure 6A), indicating that antibody was not delivered into cells by the sandwich method. Three-dimensional reconstruction of the epithelial sections confirmed that FITC signals were located above the nuclear layer (Figure 6B). It is likely that the extracellular matrix, including the thin cuticle, blocked the penetration of antibodies even with L17E. We then introduced a blocking procedure, but no delivery improvement was detected; FITC signals were located outside the epithelial cells (*n_p_* = 0; *n_t_* = 3) (Figure 6C), which was confirmed by three-dimensional reconstruction of the epithelium (Figure 6D). These results showed that antibody was not delivered into the epithelial cells using the sandwich method even with L17E.

#### 3.2.2. Injection Method

**Figure 7:**
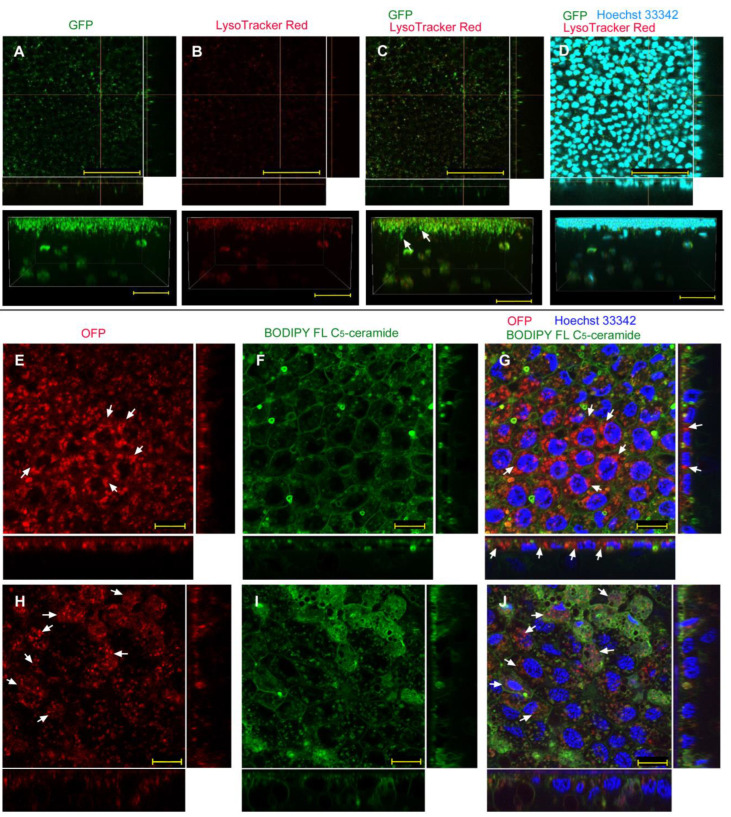
GFP/OFP application using the injection method with L17E. (**A**–**D**) A treated individual. Four panels (**A**–**D**) are images of the same visual field. Optical cross sections are shown at the right and bottom of the major plane panel. In addition, a side view of a three-dimensional reconstruction image (apical side up) is shown at the bottom. Scale bars: 50 μm. (**A**) GFP. (**B**) LysoTracker Red. (**C**) Merge of GFP and LysoTracker Red. Arrows indicate GFP-positive signals without overlap with LysoTracker Red. (**D**) Merged GFP, LysoTracker Red, and Hoechst 33342 images. (**E**–**G**) Another treated individual. Three panels (**E**–**G**) are images of the same visual field. Arrows indicate diffuse OFP signals. Scale bars: 10 μm. (**E**) OFP. (**F**) BODIPY FL C_5_-ceramide. (**G**) Merge of OFP, BODIPY FL C_5_-ceramide, and Hoechst 33342. (**H**–**J**) Another treated individual. Three panels (**H**–**J**) are images of the same visual field. Arrows indicate diffuse OFP signals. (**H**) OFP. (**I**) BODIPY FL C_5_-ceramide. (**J**) Merge of OFP, BODIPY FL C_5_-ceramide, and Hoechst 33342.

With L17E for injection, GFP appeared to be delivered into epithelial cells (*n_p_* = 7; *n_t_* = 7) (Figure 7A). GFP signals were mostly dotted. LysoTracker Red indicated that there were many lysosomes in the epithelium (Figure 7B), possibly in response to GFP injection. Most GFP and LysoTracker signals overlapped with each other, but there seemed to be some nonoverlapping signals (Figure 7C). Nuclear staining with Hoechst 33342 indicated that GFP signals were located in the same layer as the nuclei (Figure 7D).

To verify the GFP results above, we used OFP and observed the staining pattern using higher magnification (*n_p_* = 3; *n_t_* = 3). In an individual (Figure 7E–G), OFP was found in dots, but diffuse red areas were also present, which may indicate a cytosolic presence (Figure 7E). BODIPY FL C_5_-ceramide staining for membranous structures indicated that there were many vesicles inside cells (Figure 7F). Some of these vesicles coincided with OFP signals outside the Hoechst 33342 nuclear staining, but some did not (Figure 7G). Indeed, some cells appeared to be stained fully red in the cytosol. In another individual (Figure 7H–J), the OFP signals were not only dotted but also spread in the cytosol (Figure 7H). BODIPY staining indicated that there were many membranous structures inside cells, some of which were likely endosomes and lysosomes (Figure 7I). A merger of these two together with nuclear Hoechst 33342 staining suggested that OFP may be present in the cytosol (Figure 7J). Therefore, GFP/OFP can be delivered into the cytosol through the injection method using L17E. Compared to the results without any delivery reagent, the L17E results may be as efficient or slightly more efficient in terms of cytosolic delivery of GFP/OFP.

**Figure 8:**
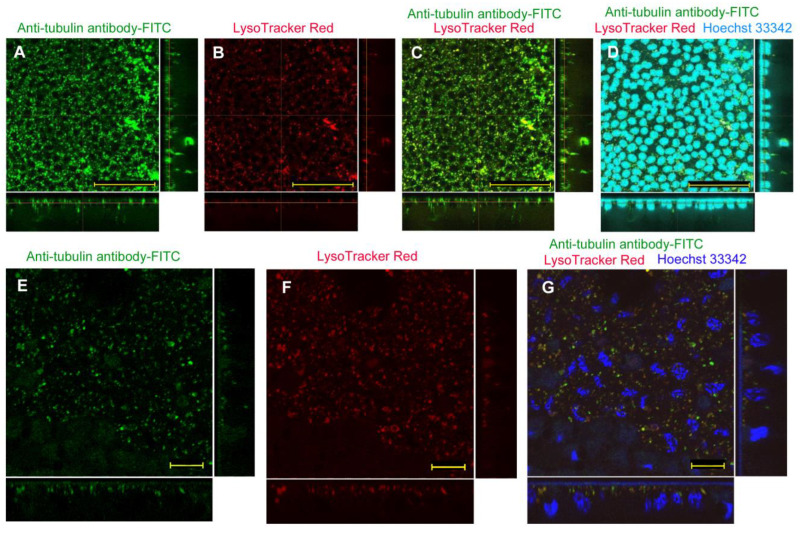
Application of anti-tubulin antibody (conjugated with FITC) using the injection method with L17E. Optical cross sections are shown at the right and bottom of the major plane panel. (**A**–**D**) A treated individual. Four panels (**A**–**D**) are images of the same visual field. Scale bars: 50 μm. (**A**) FITC. (**B**) LysoTracker Red. (**C**) Merge of FITC and LysoTracker Red, (**D**) Merge of FITC, LysoTracker Red, and Hoechst 33342. (**E**–**G**) Another treated individual. Three panels (**E**–**G**) are images of the same visual field. Scale bars: 10 μm. (**E**) FITC. (**F**) LysoTracker Red. (**G**) Merge of FITC, LysoTracker Red, and Hoechst 33342.

For the injection method using an antibody, we used anti-tubulin antibody (labeled with FITC) with L17E (*n_p_* = 20; *n_t_* = 25). Numerous FITC signals from the anti-tubulin antibody were detected in cells (Figure 8A). Numerous LysoTracker Red signals were also detected (Figure 8B). There seemed to be extensive development of lysosomes. Some antibody molecules may have been transferred to the cytosol, but most, if not all, FITC signals overlapped with LysoTracker Red signals (Figure 8C). Nuclear staining with Hoechst 33342 confirmed that FITC-free areas were mostly occupied by nuclei (Figure 8D). Higher magnification images in a different individual confirmed dotted signals from FITC (Figure 8E) and LysoTracker Red (*n_p_* = 2; *n_t_* = 3) (Figure 8F). The FITC-free areas were stained with Hoechst 33342 for nuclei (Figure 8G). We did not clearly detect diffuse FITC-positive signals from the cytosolic side despite numerous signals from dotted structures. We also employed anti-HSP antibody (*n_p_* = 4; *n_t_* = 7) and anti-*Drosophila* axons antibody (*n_p_* = 8; *n_t_* = 9) and obtained similar negative results, suggesting that the negative findings above were not antibody specific.

### 3.3. Protein Delivery with ProteoCarry Delivery Reagent

#### 3.3.1. Sandwich Method

**Figure 9:**
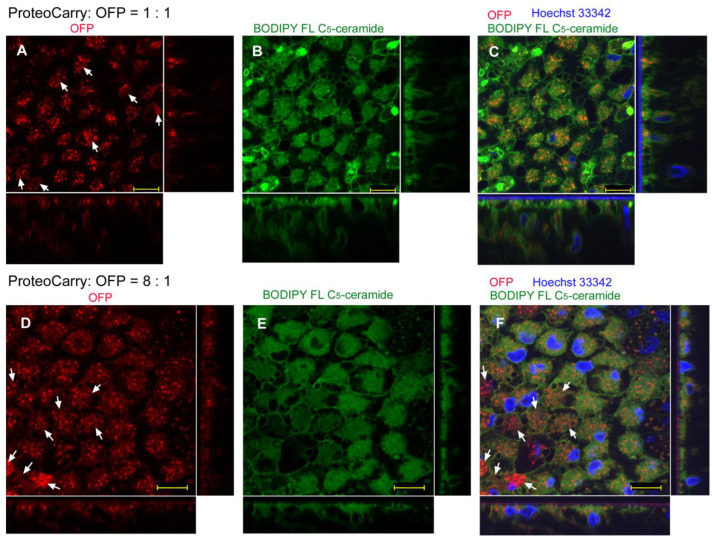
GFP/OFP application using the sandwich method with ProteoCarry. Optical cross sections are shown at the right and bottom of the major plane panel. Arrows indicate diffused OFP signals. Scale bars: 10 μm. (**A**–**C**) A treated individual. Three panels (**A**–**C**) are images of the same visual field. ProteoCarry and OFP were used in a 1:1 ratio. (**A**) OFP. (**B**) BODIPY FL C_5_-ceramide. (**C**) Merge of OFP, LysoTracker Red, and Hoechst 33342. (**D**–**F**) Another OFP-treated individual. Three panels (**D**–**F**) are images of the same visual field. ProteoCarry and OFP were used in an 8:1 ratio. (**D**) OFP. (**E**) BODIPY FL C_5_-ceramide. (**F**) Merge of OFP, BODIPY FL C_5_-ceramide, and Hoechst 33342.

We then employed another delivery reagent, ProteoCarry, with the sandwich method. Here, we concentrated on OFP together with BODIPY FL C_5_-ceramide (without GFP and LysoTracker Red) because BODIPY FL C_5_-ceramide likely stained vesicles inside cells and because GFP and OFP were expected to behave similarly. We used volumetric ratios of ProteoCarry to OFP of 1:1 (Figure 9A–C), 8:1 (Figure 9D–F), and others (see Materials and Methods).

At the 1:1 ratio, many OFP signals appeared to be confined in dotted structures, likely in endosomes and lysosomes (np = 2; nt = 2) (Figure 9A), suggesting that these OFP molecules were not transferred into the cytosol. However, there seemed to be diffuse OFP signals in the cytosol, even at a 1:1 ratio (Figure 9A). BODIPY staining revealed that cells developed an intracellular membranous network (Figure 9B). A merge of these two signals and nuclear staining with Hoechst 33342 indicated that many OFP molecules may have been in cellular vesicles (Figure 9C).

At the 8:1 ratio, OFP signals were still found in vesicular dots, but diffuse OFP signals were also observed in the cytosol of most cells to the point that cellular shapes were readily seen (*n_p_* = 4; *n_t_* = 4) (Figure 9D). Indeed, cellular outlines and intracellular membranous structures were revealed with BODIPY (Figure 9E), which were mostly similar to the OFP-positive areas in Figure 9D. Their merge, together with Hoechst 33342 nuclear staining, revealed that OFP signals were not always confined in vesicles (Figure 9F). That is, there were likely many cytosolic OFP molecules. It seemed that the 8:1 ratio produced better results than the 1:1 ratio. Additional ratios with more ProteoCarry that we tried (1.26:1, 4:1, 12:1, and 20:1) did not improve the results further.

**Figure 10:**
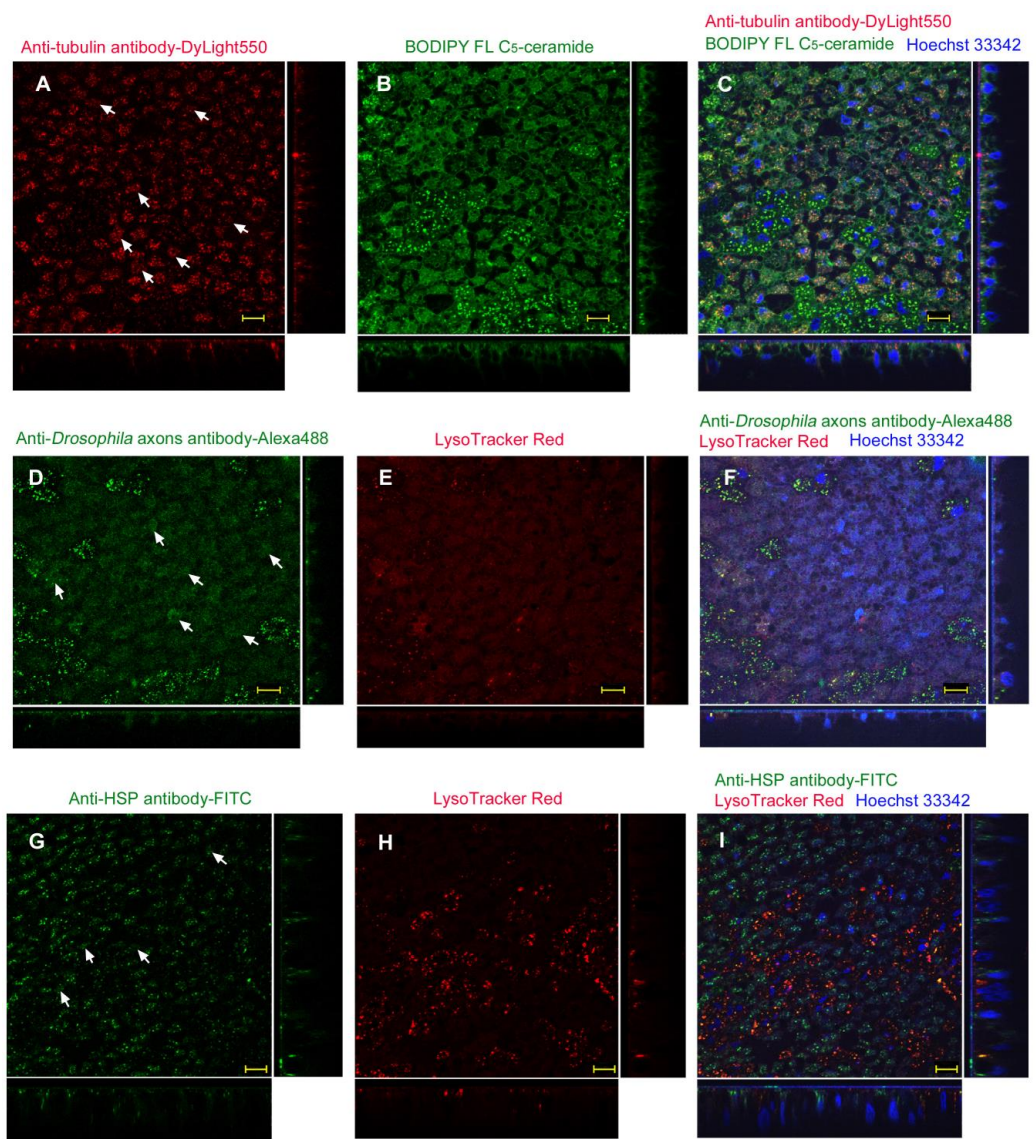
Application of antibodies using the sandwich method with ProteoCarry. ProteoCarry and antibody were used in an 8:1 ratio. Optical cross sections are shown at the right and bottom of the major plane panel. Scale bars: 10 μm. (**A**–**C**) A treated individual. Three panels (**A**–**C**) are images of the same visual field. (**A**) Anti-tubulin antibody conjugated with DyLight 550. Arrows indicate diffuse DyLight 550 signals. (**B**) BODIPY FL C_5_-ceramide. (**C**) Merge of DyLight 550, BODIPY FL C_5_-ceramide, and Hoechst 33342. (**D**–**F**) Another treated individual. Three panels (**D**–**F**) are images of the same visual field. (**D**) Anti-*Drosophila* axons antibodies conjugated with Alexa Fluor 488. Arrows indicate diffuse Alexa Fluor 488 signals. (**E**) LysoTracker Red. (**F**) Merge of Alexa Fluor 488, LysoTracker Red, and Hoechst 33342. Arrows indicate spreading cytosolic staining of Alexa Fluor 488. (**G**–**I**) Another treated individual. Three panels (**G**–**I**) are images of the same visual field. (**G**) Anti-HSP antibody conjugated with FITC. (**H**) LysoTracker Red. (**I**) Merge of FITC, LysoTracker Red, and Hoechst 33342. Arrows indicate diffuse FITC signals.

Afterward, we used the 8:1 ratio when using ProteoCarry. To examine the possibility of antibody delivery with ProteoCarry in the sandwich method, we used the following three antibodies: anti-tubulin antibody (labeled with DyLight 550) (*n_p_* = 4; *n_t_* = 4) (Figure 10A–C), anti-*Drosophila* axons antibody (labeled with Alexa Fluor 488) (*n_p_* = 3; *n_t_* = 3) (Figure 10D–F), and anti-HSP antibody (labeled with FITC) (*n_p_* = 3; *n_t_* = 7) (Figure 10G–I). To our surprise, anti-tubulin antibody was incorporated into cells via the sandwich method using ProteoCarry (Figure 10A), which contrasts with the sandwich results with L17E and with no reagent. The DyLight 550 fluorescent signals from this antibody were mostly dotted, suggesting that most antibody molecules were confined in endosomes and lysosomes. BODIPY staining revealed cellular morphology and intracellular vesicles (Figure 10B). Their merged image, together with nuclear Hoechst 33342 staining, revealed that not all vesicles overlapped with the DyLight 550 signals (Figure 10C).

The anti-*Drosophila* axons antibody also showed dotted signals from Alexa Fluor 488 (Figure 10D). In addition, the cytosolic portions were vaguely stained, suggesting that antibody molecules may escape from endosomes or lysosomes. LysoTracker Red staining showed only a small number of lysosomes (Figure 10E), consistent with the cytosolic signals from Alexa Fluor 488. A merged image with nuclear Hoechst 33342 staining revealed that only a portion of Alexa Fluor 488 and LysoTracker Red signals overlapped (Figure 10F). Most likely, most endosomes with the antibody were ruptured by ProteoCarry.

The anti-HSP antibody (labeled with FITC) showed numerous dotted signals, but also diffuse cytosolic signals (Figure 10G). LysoTracker Red also showed dotted signals (Figure 10H). Their merged image revealed that not all FITC signals overlapped with LysoTracker Red signals (Figure 10I). Overall, all three antibodies showed not only small dots that did not overlap with LysoTracker Red or BODIPY FL C_5_-ceramide but also diffuse cytosolic signals, indicating that ProteoCarry successfully released antibodies from endosomes to the cytosol in epithelial cells.

#### 3.3.2. Injection Method

**Figure 11:**
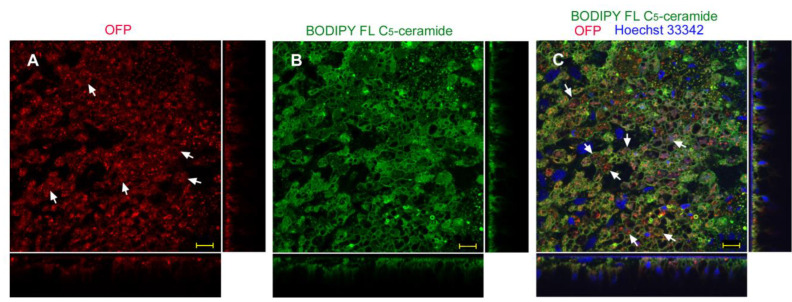
OFP application using the injection method with ProteoCarry. ProteoCarry and OFP were used in an 8:1 ratio. Optical cross sections are shown at the right and bottom of the major plane panel. All three panels show a single treated individual in the same visual field. Arrows indicate diffuse OFP signals. Scale bars: 10 μm. (**A**) OFP. (**B**) BODIPY FL C_5_-ceramide. (**C**) Merge of OFP, BODIPY FL C_5_-ceramide, and Hoechst 33342.

The results of the injection method using ProteoCarry were similar to those of the sandwich method. Numerous OFP signals were observed as dots and diffuse cytosolic staining in epithelial cells (*n_p_* = 3; *n_t_* = 4) (Figure 11A). BODIPY FL C_5_-ceramide revealed that the intracellular membrane network was well developed (Figure 11B). Their merged image with nuclear Hoechst 33342 staining revealed that OFP signals were not always in vesicles (Figure 11C). It appeared that OFP was delivered to the cytosol in some cells. We also used GFP and LysoTracker Red, the results of which were similar to those of OFP (*n_p_* = 3; *n_t_* = 3).

**Figure 12:**
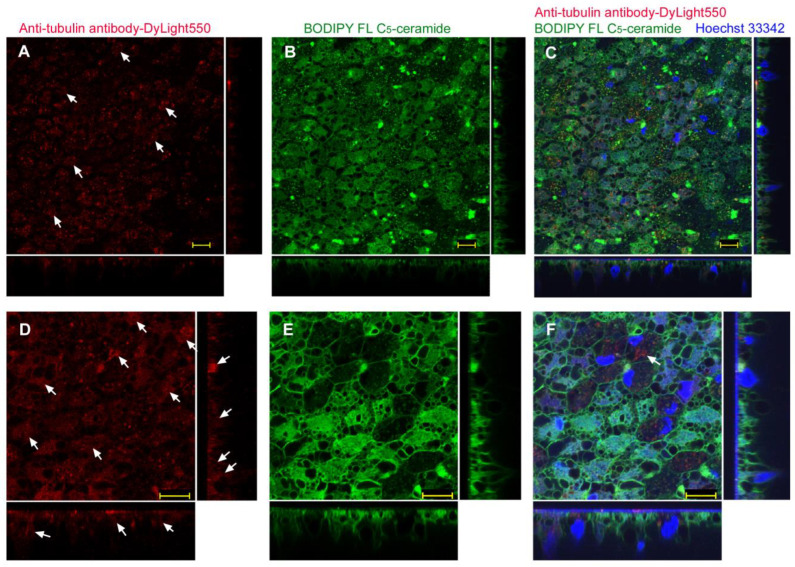
Application of anti-tubulin antibody conjugated with DyLight 550 using the injection method with ProteoCarry. ProteoCarry and antibody were used in an 8:1 ratio. Optical cross sections are shown at the right and bottom of the major plane panel. Arrows indicate diffuse or potentially structured DyLight 550 signals. Scale bars: 10 μm. (**A**–**C**) A single treated individual. Three panels (**A**–**C**) are images of the same visual field. (**A**) Anti-tubulin antibody conjugated with DyLight 550. (**B**) BODIPY FL C_5_-ceramide. (**C**) Merge of DyLight 550, BODIPY FL C_5_-ceramide, and Hoechst 33342. (**D**–**F**) Another individual. Three panels (**D**–**F**) are images of the same visual field. (**D**) DyLight 550. (**E**) BODIPY FL C_5_-ceramide (**F**) DyLight 550, BODIPY FL C_5_-ceramide, and Hoechst 33342. An arrow indicates a potential structured DyLight 550 signal with this antibody.

Injection of anti-tubulin antibody (labeled with DyLight 550) with ProteoCarry also showed dotted and diffuse DyLight 550 signals (*n_p_* = 3; *n_t_* = 4) (Figure 12A). It seemed that some antibody molecules appeared to be in the cytosol because DyLight 550 signals were diffused in cells. The BODIPY signals mostly showed small dots and intracellular membranous structures (Figure 12B). These merged images, together with Hoechst 33342 staining for nuclei, revealed that OFP-positive and BODIPY-positive vesicles did not always overlap (Figure 12C). It was difficult to observe cytosolic DyLight 550 signals in the merged image (Figure 12C), despite them appearing to spread in the DyLight 550 image (Figure 12A). This is likely because of the extensive intracellular membrane network in these cells. Another individual with higher magnification images clearly showed diffuse intracellular signals from DyLight 550 (Figure 12D). Intracellular staining was also observed via BODIPY staining (Figure 12E). A merged image blurred the diffuse OFP signals (Figure 12F).

## 4. Discussion

In the present study, we examined whether foreign proteins were translocated into butterfly wing epithelial cells in vivo, with or without a membrane-lytic peptide. The results are summarized in Table 1. We took advantage of the real-time in vivo imaging system of the butterfly wing epithelium, in which protein fluorescent signals were readily observable. We used two kinds of proteins (GFP/OFP and antibodies), two membrane-lytic peptides (L17E and ProteoCarry), and two application methods (the sandwich method and the injection method). In this way, we examined three important factors for protein delivery (i.e., protein species, delivery reagent, and application method).

In the present study, rigorous quantification of protein delivery was not possible. This is an inherent disadvantage of an in vivo system, because delivery efficiency varies among treated individuals. For some methods used in this study, only less than half of treated individuals showed observable fluorescent signals. We continued treatments until we obtained at least two well-stained individuals per method. Moreover, not all epithelial cells were stained well. Interpretable fluorescent signals were often located in clusters of cells. In addition, the detection of cytosolic staining is often more difficult than that of endosomes or lysosomes because fluorescent signals are diffused in the cytosol, resulting in relatively low fluorescence intensity (also see Materials and Methods for additional points). Nonetheless, with the limitations mentioned above, we were able to qualitatively estimate whether a protein of interest was delivered just to endosomes and lysosomes, or to the cytosol.

To our surprise, using the sandwich method, we discovered that GFP/OFP was likely taken up by cells via endocytosis without any delivery reagent. Furthermore, at least some GFP/OFP molecules appeared to be delivered into the cytosol. This delivery may be due to a unique feature of developing insect cells that have active endocytosis [35]. Such active endocytosis may be due to autocrine, paracrine, or endocrine communication among developing cells. However, this method of protein delivery appeared to be limited to GFP/OFP. Antibody delivery was not achieved at all in the sandwich method without any delivery reagent. These results are probably due to the size of these proteins; antibodies may be too large to cross the cuticle layer, the extracellular matrix, or the plasma membrane, without any delivery reagent. The molecular weights of GFP/OFP and antibodies are approximately 27 kDa and 160 kDa, respectively, showing a six-fold difference in size. In this sense, single-domain antibodies (also called nanobodies or VHH antibodies) would be expected to be delivered into the cytosol more efficiently because of their smaller size, 12–15 kDa [55].

As an alternative method, the injection method was performed, which likely delivered GFP/OFP into cells via endocytosis. We discovered that the injected GFP/OFP molecules were translocated from the basal side to the apical side of the epithelium in hemolymph and were taken up by cells from the apical side. This suggests that polarized cells, such as butterfly wing epithelial cells, may undergo active endocytosis only at the apical side. This result is consistent with the presence of the apical extracellular space that was demonstrated to be functional [51,56,57]. We also observed relatively high levels of endosomes or lysosomes after the injection of a protein of interest. Endocytosis may be promoted as a part of the immune response to a challenge of foreign proteins. However, antibodies were not delivered well without a delivery reagent, even with the injection method. Again, this could have been also because of the relatively large molecular size of antibodies.

We used two protein delivery reagents, L17E and ProteoCarry, both of which are now commercially available. L17E did not seem to improve the delivery efficacy of GFP/OFP very much using the sandwich method, although our method was not quantitative. Antibodies were not delivered at all. Improvements in delivery efficiency seemed to be small, even in the injection method. Most protein molecules were likely found in endosomes or lysosomes, although a small portion of them may have been translocated into the cytosol. Thus, roughly speaking, L17E was as efficient as or slightly more efficient than the no-reagent method.

ProteoCarry appeared to be more powerful than L17E in our system, at least when used at the 8:1 ratio. Using ProteoCarry, both in the sandwich and injection methods, both GFP/OFP and antibodies were delivered. To our surprise, ProteoCarry successfully delivered antibodies via the sandwich method. ProteoCarry may be able to break through extracellular matrices to deliver relatively large proteins such as antibodies. This topical application is methodologically advantageous over systemic application when target cells are known, because the injection method potentially delivers proteins into many cells in various tissues but simultaneously potentially damages nontarget cells due to side effects. Notably, only ProteoCarry was able to deliver antibodies into the cytosol. Unfortunately, we were not able to clearly detect specific binding patterns of antibodies to intracellular components. Among the antibodies used in this study, anti-tubulin antibodies may have interacted with α-tubulin molecules. Higher-resolution fluorescent images of anti-tubulin antibodies in other studies suggest perinuclear and mesh-like intracellular structures [58,59].

Injection with ProteoCarry did not seem to induce protein delivery into cells from nonapical sides. Interestingly, not only topical application but also systemic application made the apical incorporation of GFP/OFP or antibodies possible. These results, together with the previous results without a delivery peptide, suggest that the activity of ProteoCarry is dependent on endocytosis. As discussed before, these results further demonstrated that there is a small extracellular space between the apical cellular membrane and the procuticle layer above, and that this extracellular space is filled with hemolymph that is drawn from the abdominal hemolymph. This interpretation is consistent with the previous finding that injected FB28, a fluorescent chemical that binds to chitin, was localized in the apical extracellular site of the wing epithelial tissue [51]. These findings illustrate the dynamic nature of the apical extracellular space, which plays an active role in the color pattern determination of butterfly wings [51,56,57]. Active endocytosis may also be required for efficient in vivo protein delivery in other systems.

Although we examined just two membrane-lytic peptides in the present study, we recommend ProteoCarry as the primary choice for an in vivo protein delivery experiment, based on the present results. To our knowledge, this study is the first demonstration of in vivo protein delivery using ProteoCarry. ProteoCarry is now commercially available and is a proprietary product of Funakoshi, Japan. The precise chemical nature of this product has not yet been published. However, functional protein delivery studies using ProteoCarry would be fruitful in the future.

In this study, we focused on butterfly epithelial cells, but similar protein delivery may be applicable to a wide variety of biological systems, especially those of insects. The delivery method of choice (either the topical or systemic method) may depend on the system of interest. For instance, the topical method may be suitable for the functional analysis of proteins in a particular tissue. In contrast, to deliver antibodies or other proteins such as protein drugs for therapeutic purposes to cells throughout the body, systemic application may be a better option. For example, the injection method may be suitable for metastatic malignant cells in mammals if they are active in endocytosis. 

We believe that the present system allows researchers to perform functional protein delivery to understand the molecular functions of delivered proteins in butterfly wing development. When an antibody against a specific protein is introduced, or when a putative morphogen is delivered, a patchy distribution of functionally altered wing scales may be expected.

## 5. Conclusions

Using butterfly wing epithelial cells in vivo, this study showed that developing insect cells may be able to accept foreign proteins with the size of GFP/OFP via topical and systemic pathways without any delivery reagent, albeit to a limited degree. This delivery is likely through endocytosis, but some molecules were probably delivered to the cytosol. The delivery process appeared to be slightly promoted by a membrane-lytic peptide, L17E. However, antibody delivery was difficult with L17E, likely because of relatively large molecular weights of antibodies. Another delivery peptide, ProteoCarry, had the ability to transfer not only GFP/OFP but also antibodies into the cytosolic side beyond endosomes via topical and systemic pathways. ProteoCarry is a peptide delivery reagent that is commercially available and practically usable for in vivo applications. The present results demonstrated that protein delivery was possible in insect epithelial cells in vivo, and will provide opportunities for functional molecular analyses of proteins in various biological systems, especially those of insects, including the butterfly wing developmental system.

## Figures and Tables

**Table 1:** Summary of protein delivery results in the present study.

	Sandwich Method		Injection Method	
Delivery Reagent	GFP, OFP	Antibodies	GFP, OFP	Antibodies
No reagent	Endosome + Cytosol (Figure 1)	No delivery (Figure 2)	Endosome + Cytosol (Figure 3)	Endosome (Figure 4)
L17E	Endosome + Cytosol (Figure 5)	No delivery (Figure 6)	Endosome + Cytosol (Figure 7)	Endosome (Figure 8)
ProteoCarry	Endosome + Cytosol (Figure 9)	Endosome + Cytosol (Figure 10)	Endosome + Cytosol (Figure 11)	Endosome + Cytosol (Figure 12)

Note: Information in this table is based on qualitative evaluations of confocal images. Endosomes and lysosomes are not differentiated, and they are indicated as “Endosome.”

## Data Availability

All data relevant to the results and conclusions are included in this study.
